# Evaluating causality of cellular senescence in non-alcoholic fatty liver disease

**DOI:** 10.1016/j.jhepr.2021.100301

**Published:** 2021-05-01

**Authors:** Abraham Stijn Meijnikman, Hilde Herrema, Torsten Pascal Marcel Scheithauer, Jeffrey Kroon, Max Nieuwdorp, Albert Kornelis Groen

**Affiliations:** 1 Department of Internal and Vascular Medicine, Amsterdam University Medical Centers, location AMC, Amsterdam, the Netherlands

**Keywords:** cellular senescence, non-alcoholic fatty liver disease, non-alcoholic steatohepatitis, senolytics, obesity, ATM, ataxia telangiectasia mutated, CDK, cyclin dependent kinase, C/EBPα, CCAAT- enhancer-binding protein, DDR, DNA damage response, FFAs, free fatty acids, HCC, hepatocellular carcinoma, IL-, interleukin, KC, Kupffer cell, LSEC, liver sinusoidal endothelial cell, MCP1/CCL2, monocyte chemoattractant protein-1, MiDAS, mitochondrial dysfunction-associated senescence, NAFL, non-alcoholic fatty liver, NAFLD, non-alcoholic fatty liver disease, NASH, non-alcoholic steatohepatitis, qPCR, quantitative PCR, Rb, retinoblastoma factor, ROS, reactive oxygen species, SA-β gal, senescence-associated beta-galactosidase, SASP, senescence-associated secretory phenotype, SCAP, senescence-associated antiapoptotic pathways, TGFβ, transforming growth factor-β, TNFα, tumour necrosis factor-α

## Abstract

Cellular senescence is a state of irreversible cell cycle arrest that has important physiological functions. However, cellular senescence is also a hallmark of ageing and has been associated with several pathological conditions. A wide range of factors including genotoxic stress, mitogens and inflammatory cytokines can induce senescence. Phenotypically, senescent cells are characterised by short telomeres, an enlarged nuclear area and damaged genomic and mitochondrial DNA. Secretion of proinflammatory proteins, also known as the senescence-associated secretory phenotype, is a characteristic of senescent cells that is thought to be the main contributor to their disease-inducing properties. In the past decade, the role of cellular senescence in the development of non-alcoholic fatty liver disease (NAFLD) and its progression towards non-alcoholic steatohepatitis (NASH) has garnered significant interest. Until recently, it was suggested that hepatocyte cellular senescence is a mere consequence of the metabolic dysregulation and inflammatory phenomena in fatty liver disease. However, recent work in rodents has suggested that senescence may be a causal factor in NAFLD development. Although causality is yet to be established in humans, current evidence suggests that targeting senescent cells has therapeutic potential for NAFLD. We aim to provide insights into the quality of the evidence supporting a causal role of cellular senescence in the development of NAFLD in rodents and humans. We will elaborate on key cellular and molecular features of senescence and discuss the efficacy and safety of novel senolytic drugs for the treatment or prevention of NAFLD.

**Key points**

• Cellular senescence has been put forward as a contributing factor in the development of NAFLD.
• Senescent cells exhibit the following 4 hallmarks: i) prolonged and generally irreversible cell cycle arrest, ii) macromolecular damage, iii) secretory features and iv) deregulated metabolism which are all present in hepatocytes of both humans and rodents with NAFLD.
• Data from studies in rodents and humans have shown that NAFLD is accompanied by an increase in senescent cells in the liver, and that the number of senescent cells is associated with disease progression.
• Under normal circumstances, around 3–7% of hepatocytes are senescent and this percentage can increase to 50–100% in end-stage liver disease.
• Only a few markers reliably detect senescent cells at this moment in time and novel, non-invasive analytical tools are needed to better understand the role of senescent cells in NAFLD.
• Rodent studies strongly point to a causal role of cellular senescence in the development of NAFLD.
• Despite the strong association between senescence and NAFLD, it remains unclear whether hepatic senescence is a consequence, a cause or both.
• Targeting senescence has emerged as an attractive therapeutic target for NAFLD since senescence might be involved in the full spectrum of the disease.
• Senolytic drugs can be administrated intermittently, thereby minimising potential toxic effects and increasing adherence in individuals who are often affected by multiple morbidities and thus treated with multiple medications.

## Introduction

Accompanying the obesity pandemic, the prevalence of non-alcoholic fatty liver disease (NAFLD) is rapidly increasing (exceeding 80% in morbidly obese individuals).^1^ NAFLD represents a spectrum of liver diseases with clinical and histological abnormalities ranging from non-alcoholic fatty liver (NAFL) in the case of isolated steatosis to non-alcoholic steatohepatitis (NASH), fibrotic NASH, advanced fibrosis, cirrhosis, and hepatocellular carcinoma (HCC).^2^ Accumulation of fat in hepatocytes has long been considered a relatively benign condition^3^^,^^4^ However, around 30% of individuals with NAFL progress to well-defined NASH with clinically significant fibrosis.[5], [6], [7] Advanced forms of NAFLD often require a liver transplantation and are a major cause of liver-related deaths.^1^ The rapidly growing prevalence of NAFLD and lack of effective treatment options to tackle this potentially debilitating disease, will further increase the obesity-related burden on public health and economies. In order to develop appropriate, non-invasive diagnostic methods and treatment options, it is critical to deeply investigate the complex pathophysiology of NAFLD.

The underlying mechanisms that govern hepatic lipid accumulation and the predisposition to inflammation and fibrosis are complex and multifactorial. In the past decades, a multitude of disease-inducing factors have been unveiled, resulting in the multi-hit hypothesis, which integrates parallel and synergistically operating disease promoting factors.^2^^,^^8^ Insulin resistance,^9^ adipocyte dysfunction,^10^ genetic variants,^11^ bile acid metabolism,^12^ the gut microbiome,^13^ and lipotoxicity^14^ have been extensively studied in relation to their roles in the pathogenesis of NAFLD. These players are unified by the metabolic dysregulation accompanying obesity. Metabolic dysregulation refers to a complex range of metabolic alterations often induced by insulin resistance. For example, insulin resistance increases circulating free fatty acid (FFA) levels by reducing insulin-mediated suppression of lipolysis in the visceral adipose tissue compartment.^15^ This increases the hepatocellular influx of FFAs which may subsequently be stored as triglycerides. Increased fat storage in the liver is strongly linked to reduced hepatic insulin sensitivity and a consequential increase in hepatic gluconeogenesis, a major contributor to the hyperglycaemia observed in diseases associated with NAFLD.^16^ Moreover, *de novo* lipogenesis is increased due to the constant high levels of insulin, producing even more triglycerides and further enhancing hepatic gluconeogenesis^17^^,^^18^ Thus, hepatic insulin resistance in individuals with NAFLD is considered to be limited to the pathway involving suppression of hepatic glucose production and not the lipogenic pathway, which is referred to as selective insulin resistanc.^19^ Accumulating evidence obtained in the past decades revealed that this pathogenic paradox plays a pivotal role in the development of NAFLD^20^^,^^21^

Triglyceride accumulation is not hepatotoxic *per se* and could even represent a defensive mechanism to counterbalance FFA excess. However, high levels of FFAs, free cholesterol and other lipid metabolites can lead to lipotoxicity.^14^ Lipotoxicity causes mitochondrial dysfunction, resulting in the formation of reactive oxygen species (ROS), endoplasmic reticulum (ER) stress, inflammation and cell damage^14^^,^^22^ As a consequence of the overload of FFAs, proinflammatory pathways are activated, leading to hepatic inflammation and eventually fibrosis.^23^^,^^24^ Expansion of subcutaneous and visceral adipose tissue compartments in obesity leads to hypoxia-induced hypersecretion of adipocytokines such as tumour necrosis factor (TNF)α, interleukin (IL)-6 and monocyte chemoattractant protein-1 (MCP-1/CCL2) by adipocytes.^25^^,^^26^ In addition, the inflammatory immune cells that accumulate in the adipose tissue of individuals with obesity further perpetuate the low-grade inflammatory state^25^^,^^26^ These proinflammatory mediators are secreted into the circulation and contribute to activation of inflammatory signalling pathways in the liver, thereby contributing to the development and progression of NAFLD.^25^^,^^26^

Recently, cellular senescence has been put forward as a contributing factor in the progression of NAFLD. Cellular senescence is one of the hallmarks of aging and is defined as a stable arrest of the cell cycle coupled to specific phenotypic changes.^27^ Senescent cells secrete a collection of proteins called the senescence-associated secretory phenotype (SASP).^28^^,^^29^ This pro-inflammatory secretome has been suggested to drive age-related tissue dysfunction. Interestingly, metabolic dysregulation is thought to favour cellular senescence in several tissues involved in the pathogenesis of NAFLD such as the liver, pancreas and adipose tissue, further perpetuating metabolic dysregulation. The number of senescent cells is particularly increased in the adipose tissue and liver of individuals with obesity.[30], [31], [32] Hepatocytic senescence has been shown to impair mitochondrial β-oxidation.^33^ Concomitantly, SASP components are abundantly present in adipose tissues of individuals with obesity and may promote insulin resistance and inflammation.^31^ Senescence has also been linked to the hyperinsulinemic state often observed in individuals with obesity. Senescence in pancreatic beta cells induces greater glucose uptake and mitochondrial activity, leading to increased insulin secretion.^34^ This observation suggests that beta cell senescence enhances the insulin-secreting capacity of the pancreas. This is in contrast to the general dogma that cellular senescence leads to a deterioration of cell and organ function.^35^ Indeed, animal studies have shown that tissue function, including liver metabolism, can be recovered by clearing senescent cells.^33^ Targeting senescence might therefore be an interesting future therapeutic option to tackle cardiometabolic diseases, including NAFLD.

In this review, we aim to provide insights into the quality of the evidence that supports a causal role of cellular senescence in the development of NAFLD in rodents and humans. We will elaborate on the key cellular and molecular features of senescence. Finally, we will discuss the effectiveness and safety of novel senolytic (senescence destroying) drugs to treat this disease.

## Hallmarks of cellular senescence

**Fig. 1:**
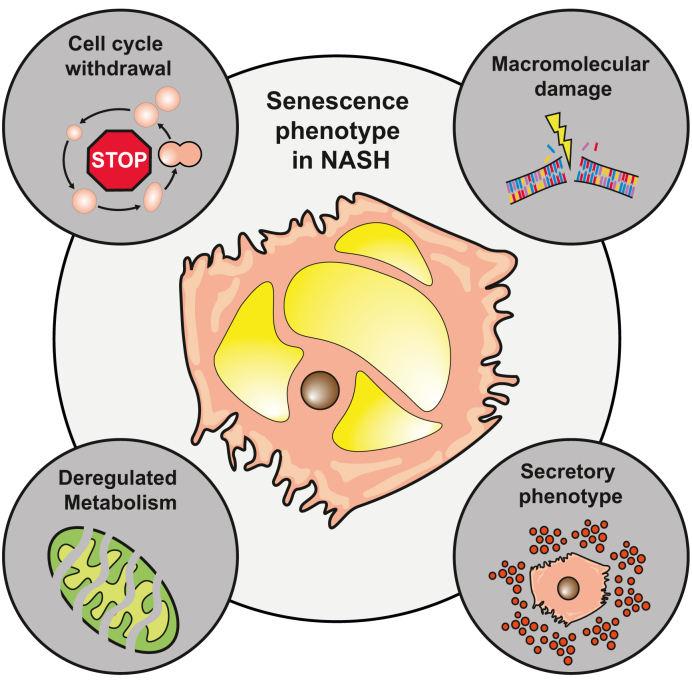
Senescent cells in general and in NASH exhibit 4 hallmarks. 1) prolonged and generally irreversible cell cycle arrest, 2) macromolecular damage, 3) secretory features and 4) deregulated metabolism. NASH, non-alcoholic steatohepatitis.

Cellular senescence, originally described by Hayflick and Moorhea,^36^ is a cellular state implicated in various physiological processes. Senescent cells exhibit the following 4 hallmarks: i) prolonged and generally irreversible cell cycle arrest, ii) macromolecular damage, iii) secretory features and iv) deregulated metabolism (Fig. 1)^37^^,^^38^ Senescence is driven by a variety of factors such as genotoxic stress, mitogens and inflammatory cytokines. Also, metabolic factors including high glucose levels, ceramides, fatty acids, prostanoids and ROS are capable of inducing cellular senescence. Furthermore, signals originating from senescent cells have been shown to be able to transfer the senescent phenotype to neighbouring cells.^39^ In addition, senescence is also linked to the age-associated loss of the regenerative capacity in the liver after severe liver injury.^40^^,^^41^ Following acute liver damage, senescence occurs in hepatocytes as well as non-parenchymal cells in the livers of adult but not young mice, leading to impaired regeneration. Recently, Ritschka *et al*^42^ showed that treatment with the senolytic drug agent ABT-737 (a BCL-2 family inhibitor) decreased markers of senescence in hepatocytes and reduced inflammation, which was associated with an improvement in liver function and regeneration following partial hepatectomy in adult mice.

### Cell cycle withdrawal and macromolecular damage

These senescence-inducing signals activate transcriptional cascades which culminate in the activation of the cyclin-dependent kinase inhibitors p21 or p16, resulting in irreversible cell cycle arres.^43^ This will eventually lead to specific phenotypic changes such as telomere shortening, nuclear area enlargement and genomic and mitochondrial DNA damag.^44^ There are 2 major mechanisms of cellular senescence; one is replicative senescence and the other is stress-induced premature senescenc.^43^^,^^45^ Replicative senescence depends on telomere shortening or erosion, predominantly upon aging, whereas stress-induced premature senescence is telomere-independent and refers to intracellular or environmental stress factors leading to macromolecular damage (*i.e.* DNA damage, protein damage, lipid damage).^43^^,^^45^^,^^46^ Both mechanisms induce a complex multigenic pathway known as the DNA damage response (DDR).^47^ The DDR can inhibit cell cycle progression and prevent the propagation of corrupted genetic information to neighbouring cell.^47^ Some factors involved in the DDR, such as the phosphorylation of histone H2AX (yH2AX) and its associated proteins, including MDC1, 53BP1 and the activated form of the kinase ataxia telangiectasia mutated (ATM), accumulate at sites of DNA damage. These factors form cytologically detectable nuclear foci and mark the individual sites of DNA damage, which subsequently contribute to checkpoint enforcement and cell cycle arrest, until damage has been repaired. If DNA damage persists, the tumour suppressor p53 will be phosphorylated, via activation of ATM, stimulating the expression of p21, an essential mediator of senescence-associated cell cycle arrest.^48^ After the early activation of p21, p16 (which is also suggested to play a role in several types of senescence) is activated. p16 inhibits cyclin-dependent kinase-4 (CDK4) and cyclin-dependent kinase-6 (CDK6), thus maintaining the senescent phenotype.^49^ Activation of either p21 or p16 results in the inhibition of retinoblastoma factor (Rb) phosphorylation, allowing it to bind to the E2F transcription factor which prevents cell division.^50^

### Senescence-associated secretory phenotype

Another hallmark of senescence is the SAS^28^^,^^29^^,^^39^ This proinflammatory secretome is a hallmark of senescent cells and contributes to tissue dysfunction in both an autocrine and paracrine fashio.^39^ Interestingly, the SASP stimulates the immune system to clear senescent cells but can also reinforce or even maintain the senescent cell state.[51], [52], [53] Furthermore, it has been suggested that the SASP contributes to persistent chronic inflammation, which is often observed in cardiometabolic diseases including NASH, and can explain some of the deleterious pro-aging effects of senescent cells.^54^^,^^55^ The SASP is regulated by various mechanisms. For example, remodelling of enhancer regions of genes results in changes in the phenotype of senescent cells and induces qualitative and quantitative changes in their secretome. In addition, transcription factors such as GATA4 (an upstream regulator of NF-kB) and mammalian target of rapamycin (mTOR) as well as p38MAPK signalling pathways have been strongly implicated in the regulation of the SASP.[56], [57], [58] Interestingly, GATA4 has been reported to be involved in autophagy, which is a highly regulated cellular programme involved in recycling of intracellular proteins and damaged/non-functional organelles.^59^ GATA4 is degraded by p62-mediated selective autophagy under normal circumstances. Interestingly, during senescence, this regulation is suppressed, which may initiate and maintain the SASP and thereby facilitate senescence.^57^ Recently, GATA6 has been implicated in the induction of senescence. When GATA6 accumulation is not decreased by autophagy, the expression of p53 and p16 is enhanced, while knocking down GATA6 reduces the upregulation of p53 and p16, and thereby hepatic senescence.^60^ *In vitro* data showed that the SASP is produced in a p16-independent manner as a result of DDR-dependent and independent signalling through p38MAPK and NF-kB. Whereas the early SASP is dominated by growth factors such as transforming growth factor-β (TGF)β which triggers senescence in an autocrine fashion,^56^^,^^61^ a switch towards a more pro-inflammatory SASP is established through the activation of NOTCH1, where secreted and membrane bound IL-1a acts in an autocrine fashion to reinforce the production of IL-6 and IL-8.^62^^,^^63^

### Dysregulated metabolism

Accumulating evidence suggests a bidirectional link between cellular senescence and mitochondria.^64^ Senescent cells are capable of deregulating metabolism by altering mitochondrial function, dynamics and morphology. In the early stages of senescence, deterioration of mitochondrial oxidative phosphorylation increases production of ROS.^65^^,^^66^ ROS can maintain and enhance senescence through feedback loops that replenish the DDR.^67^^,^^68^ Of interest, mitochondrial DNA is highly vulnerable to ROS due to its close proximity to the generation site, while damage to mitochondrial DNA further impairs oxidative phosphorylation. Several DNA repair mechanisms exist within a cell to restore DNA integrity. While these pathways have been extensively studied in the nucleus, current knowledge on, and evidence for, DNA repair pathways in the mitochondria are more limited.^69^ Furthermore, mitochondrial ROS accelerates telomere shortening and triggers senescence in a paracrine fashion^68^^,^^70^ *In vitro* data have shown that senescent cells induce considerable metabolic changes on the cellular level, related to mitochondrial metabolites (*i.e.* decrease in NAD+/NADH ratio and tricarboxylic acid cycle metabolites^68^^,^^71^). Also, changes in mitochondrial dynamics such as biogenesis, fusion, fission and mitophagy have been described in senescent cells.^67^^,^^72^ Interestingly, mitochondrial dysfunction was recently shown to induce a distinct type of senescence termed MiDAS (mitochondrial dysfunction-associated senescence), as a result of a decreased NAD+/NADH rati.^73^ The authors showed that altered AMP/ATP and ADP/ATP ratios activate AMPK which may induce senescence by phosphorylating p53 or stabilising p1.^73^

The aforementioned hallmarks of cellular senescence are observed in hepatocytes of both humans and rodents with NAFLD. Nevertheless, it has been suggested that hepatocytic senescence is a mere consequence of the metabolic dysregulation and inflammatory phenomena observed in fatty liver disease instead of a causal player. This chicken-egg situation can be clarified in large, prospective studies, which will provide insight into the timeline of disease development linked to the presence of cellular senescence. A causal contribution (*i.e*., cellular senescence as a driving factor for disease development) can only be substantiated from results of highly targeted interventional studies. To successfully identify, characterise and pharmacologically eliminate senescent cells, one of the major limitations of the field needs to be overcome: robust, cell-and pathway-specific biomarkers for cellular senescence need to be developed. Driven by the non-specificity of many current senescence markers and the existence of distinct senescence programmes, the scientific community has struggled to identify universal and unequivocal signatures that characterise the senescent stat.^74^

## Markers and detection of cellular senescence

The development and optimisation of sensitive and specific assays to track senescent cells is challenging because of the complex and cell-specific senescent phenotypes. Of importance, numerous non-senescent cells, especially proinflammatory cells such as macrophages and (pre-)cancerous cells, share features with senescent cells and impair specificity of currently available assays.^75^ Hence, only a few markers reliably detect senescent cells at this moment in time and novel (combined) analytical tools are urgently needed to better understand the role of senescent cells in NAFLD.

The first tool to successfully identify senescent cells arose from observations that senescent cells display β-galactosidase enzymatic activity at pH 6, whereas more common β-galactosidase isoforms show peak enzymatic activity at pH 4–4.5. This is referred to as senescence-associated β-galactosidase (SA-β gal).^76^^,^^77^ Shortly thereafter, the cyclin-dependent kinase inhibitor p16, which serves as a master regulator of cell cycle arrest, was shown to play a role in senescenc.^78^ In the past decade, numerous other senescence markers such as increased cell size and intracellular protein content, accumulation of lipofuscin, increased expression of p21, epigenetic profiles and SASP factors have been identified and linked to distinct senescence pathway.^39^^,^^74^ An overview of currently used markers of senescence is provided in Table 1.

**Table 1:** **Overview of senescence markers**.

Senescent cell hallmark	Class	Markers
Cell cycle arrest	Lack of DNA synthesis	BrdU, EdU
	Lack of proliferation	Ki67
	Activation of p16-pRB axis	p16INK4a, pRB, phospho-pRb
	Activation of p53-p21 axis	p21, p53, phospho-p53, DEC1 (BHLHB2), PPP1A
Structural changes	Morphology, cell size	Morphology, cell size
	Increased lysosomal compartment and activity	SA-β-galactosidase, SA-α-Fucosidase, Lipofuscin
	DNA damage	γH2AX, 53BPI, Rad17, ATR, ATM, MDC1, TIF.
	Telomere shortening	Telomeres
	SAHFs formation	DAPI/Hoechst 33342, HIRA, H3K9-methylation, PML bodies, HP1-gamma
	Nuclear membrane	Lamin B1
Pro-survival	Apoptosis exclusion	Annexin V, BCL-2, Cleaved PARP, Cleaved caspase 2/3/9, TUNEL staining
SASP	Chemokines	IL-8; GRO-a, -b, -g; MCP-2; MCP-4; MIP-1a; MIP-3a; HCC-4; eotaxin; eotaxin-3; TECK; ENA-78; I-309; I-TAC
	Growth factors; regulators	Amphiregulin; epiregulin; heregulin; EGF; bFGF; HGF; KGF (FGF7); VEGF; angiogenin; SCF; SDF-1; TGFb; PIGF; NGF; IGFBP-2, -3, -4, -6, -7
	Insoluble factors	Amphiregulin; epiregulin; heregulin; EGF; bFGF; HGF; KGF (FGF7); VEGF; angiogenin; SCF; SDF-1; TGFb; PIGF; NGF; IGFBP-2, -3, -4, -6, -7
	Interleukins	IL-6; IL-7; IL-1; IL-1b; IL-13; IL-15
	Non-protein molecules	PGE2; nitric oxide; ROS
	Other inflammatory molecules	GM-CSE; G-CSE; IFN-g; BLC; MIF
	Proteases and regulators	MMP-1, -3, -10, -12, -13, -14; TIMP-1; TIMP-2; PAI-1, -2; tPA; uPA; cathepsin B
	Receptors; ligands	ICAM-1, -3; OPG; sTNFRI; sTNFRII; TRAIL-R3; Fas; uPAR; SGP130; EGF-R

SASP, senescence-associated secretory phenotype.

**Fig. 2:**
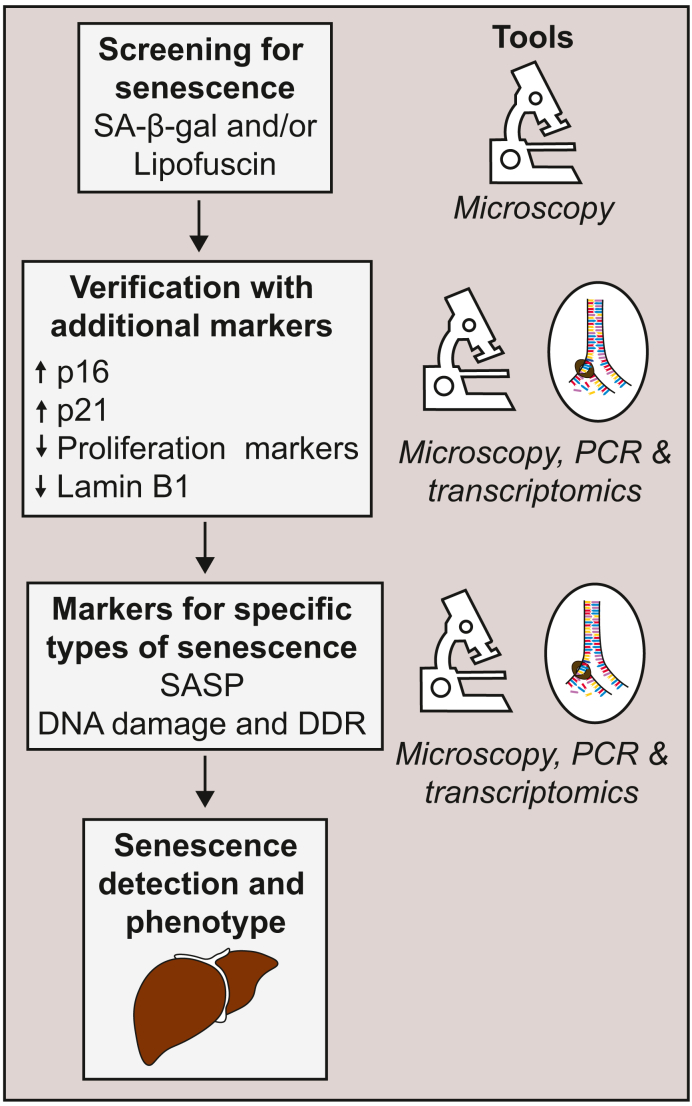
The proposed multi-marker workflow approach.^74^ Cells would first be screened for SA-β gal or lipofuscin staining. Initial senescence leads would then be verified by additional markers such as p16 or p21 and further specified into specific types of senescence by characterizing SASP or DDR. For the detection of the senescent cells several tools can be used such as immunohistochemistry, qPCR or transcriptomic analyses. DDR, DNA damage response; qPCR, quantitative PCR; SA-β gal, senescence-associated beta-galactosidase; SASP, senescence-associated secretory phenotype.

Unfortunately, these markers have their limitations. For example, SA-β gal activity can be high in macrophages and even p16, which is considered to be one of the most specific senescence markers, is also expressed in certain non-senescent cells.^75^^,^^79^ Moreover, p16 is not expressed by all senescent cells. To overcome these challenges, a multi-marker approach based on immunohistochemistry and quantitative PCR (qPCR) or transcriptomic analyses was proposed to identify senescent cell.^74^ Cells would first be screened for SA-β gal or lipofuscin staining. Initial senescence leads would then be verified by additional markers such as p16 or p21 and further specified into specific types of senescence by characterising SASP or DDR (Fig. 2).^74^

This multi-marker approach enables the detection of senescent cells in various experimental settings and tissues. Promising results have been achieved in the first clinical trials targeting cellular senescence in humans^80^^,^^81^ Therefore, it is of critical importance to develop, implement, test, and harmonise methods and standard operating protocols for translational early phase trials of agents that target fundamental aging processes. New, effective and low-cost assays are needed to detect and trace senescence in blood, cells and biopsies of the targeted organ for use in clinical trials. Circulating microvesicles originating from senescent cells, senescence-specific micro-RNAs or epigenetic profiles are currently being evaluated as novel composite assays to detect senescence.^82^ Of interest, recently, Saif *et al.*^83^ were able to non-invasively measure the accumulation of lipofuscin in the liver using near infrared and shortwave infrared autofluorescence. This technique might serve as a diagnostic medical tool or could be used in clinical trials targeting senescence in the liver. Several ongoing studies are aiming to discover novel markers using “omics” techniques to quantify various macromolecules, even at the single cell level (to account for intrapopulation variability).^74^

## Insights obtained from studies in rodents on the role of senescence in NAFLD

Hepatocytic senescence can induce remarkable changes in tissue homeostasis and the hepatic microenvironment. Under normal circumstances, hepatocytes are considered to be reversed post-mitotic cells that preserve their proliferating potential. Yet, natural aging correlates with loss of proliferating potential, functionality and thus regenerative capacity of the hepatocyte.^84^ Of interest, cellular senescence is considered one of the hallmarks of aging^27^ and indeed aging itself is a major risk factor for NAFLD development and progression.^43^ Increased oxidative stress and age-related mitochondrial dysfunction have been shown to contribute to NAFLD development in old mice fed a high-fat diet (HFD).^85^ Indeed when young and old mice were treated with the same profibrotic regimen, old mice developed more severe fibrosis in their liver compared to their younger counterparts.^86^^,^^87^ Also, aging is associated with upregulation of CDK4 in the liver. CDK4 phosphorylates CCAAT- enhancer-binding protein (C/EBPα) which facilitates formation of C/EBPa-p300 complexes leading to NAFLD in the presence of a metabolic driver (*i.e.* overnutrition). *Vice versa*, pharmacological inhibition of CDK4 reduces NAFLD.^88^^,^^89^

Age-independent hepatocytic senescence has also been described in rodents. In 2000, Rudolph and DePinho revealed that progressive and repetitive liver damage in mice induces hepatocytic cellular senescence and subsequently cirrhosis.^90^ Other studies in rodents have shown a clear relation between senescence, NAFLD and liver fibrosis. Obesity-prone rats fed a HFD developed NAFLD after 13 weeks with a concomitant increase in hepatic gene expression of p16 and p21 compared to age-matched lean controls. Subsequently, the increased p16 and p21 resulted in a significant decrease in the phosphorylation of retinoblastoma protein (Rb), thereby inducing cell cycle arrest.^91^ p53-deficient mice fed a methionine- and choline-deficient diet (a widely used diet to induce NAFLD in rodents), had slower disease progression compared to wild-type mice.^92^

Although previous studies have provided insights into the putative role of cellular senescence in the development of NAFLD, data pointing towards causality was only recently published by Ogrodnik *et al..*^33^ First, NAFLD was associated with several markers of senescence in hepatocytes, such as increased senescence-associated damage foci, as determined by the presence of yH2AX, increased senescence-associated distention of satellites and larger nuclear areas.^33^ Second, hepatocytic senescence was shown to impair hepatic mitochondrial β-oxidation, thereby hindering fatty acid elimination and promoting triglyceride accumulation.^33^ Finally, a causal link between hepatocytic senescence and hepatic steatosis was unravelled using INK-ATTACK mice and a senolytic drug cocktail. INK-ATTACK transgenic mice (INK-linked apoptosis through targeted activation of caspase) contain an inducible suicide gene in the *CDKNA2* locus, which encodes p16, a key molecule in senescent cells.^93^ By using this elegant rodent model, it is possible to selectively eliminate p16-expressing cells *in vivo* following the administration of a specific molecule that dimerises the FKBP-CASP8 fusion protein and induces apoptosis. Following the systemic clearance of p16-expressing cells, hepatocytic senescence decreased, which was accompanied by the amelioration of hepatic steatosis. The administration of the senolytic drug cocktail dasatinib and quercetin to *db/db* mice reduced the number of senescent cells and led to clearance of triglycerides from the hepatocytes.^33^ However, it is important to note that the authors did not account for the possible pleiotropic effects of the senolytic drug cocktail. Moreover, to what extent these results can be translated to the entire spectrum of NAFLD (*i.e.* hepatocyte ballooning, lobular inflammation and fibrosis) remains to be investigated.

Epigenetic modifications have been observed in senescent cells. These modifications resemble a DNA methylation profile similar to those observed in cancer and aging.^94^^,^^95^ A global loss of DNA methylation at CpG sites is characteristic of replicative senescence. This loss is followed by a focal increase in DNA methylation at certain CpG islands^95^^,^^96^ Senescence-associated DNA methylation patterns have been shown to alter expression of genes typically involved in hepatic lipid metabolism in mice fed a choline- and folate-deficient diet.^97^ Alterations in the methylome profile of hepatocytes could therefore determine the severity of NAFLD. Individual histone modifications are also altered during senescence and NAFLD.^96^ Certain histone modifications such as elevation of H4K20me3 and H3K9me3 are in fact crucial for induction of proliferation arrest^96^^,^^98^ whereas elevation of H3K27ac in gene enhancers promotes the SASP.^74^ Increased expression of p21 was associated with increased acetylation of both histone H3 and H4, while decreased trimethylation of H3K27 at the p21 promotor was observe.^91^

## Senescence beyond the hepatocyte

Importantly, senescence in the liver is not limited to hepatocytes. For example, obesity-associated senescence has been observed in hepatic stellate cells (HSCs).^99^ In healthy livers, HSCs are in a quiescent state; they are activated following liver injury and play an important role in liver fibrosis. Indeed, liver fibrosis is accompanied by the excessive deposition of extracellular matrix by HSCs. Interestingly, when HSCs become senescent, they can limit the extent of fibrosis. Indeed, HSCs deficient in the key senescence genes p53 or Rb continued to proliferate and contributed to excessive extracellular matrix deposition. These findings suggest that senescence in HSCs could be beneficial. Moreover, senescent HSCs secrete matrix metalloproteases that digest matrix metalloproteins and collagens such as CollA. In contrast, Yoshimoto and colleague^99^ showed in a mouse model that the gut microbial metabolite deoxycholic acid, a metabolite that has been associated with insulin resistance and NAFLD provokes the SASP phenotype in HSC.^12^^,^^100^ This phenotype subsequently facilitates the development of HCC via the secretion of inflammatory and tumour-promoting factor.^99^ Moreover, fibroblasts and non-tumoral HSCs demonstrated increased expression of senescence and SASP markers in NASH-related HCC compared to HCCs of other aetiology.^101^ However, patients with NASH-HCC were significantly older, had a higher BMI and more metabolic diseases such as diabetes compared to patients with other aetiologies of HCC, thus these data should be interpreted with caution. Therefore, more in-depth studies are needed to understand the balance between fibrogenic and non-fibrogenic SASP in senescent HSC.

Senescence has also been observed in cholangiocytes and may have potential deleterious effects in biliary diseases, such as primary biliary cholangitis and primary sclerosing cholangitis.^102^^,^^103^ In addition, cholangiocyte senescence has also been demonstrated in other chronic parenchymal liver diseases including NAFLD.^103^^,^^104^ Recently, an *in vivo* model was introduced to study the detailed mechanism of cholangiocyte-mediated biliary senescence in the liver.^105^ The activation of senescence in cholangiocytes induced profound alterations in the cellular and signalling microenvironment, resulting in the deposition of collagen and TGFβ production and the induction of senescence in neighbouring cholangiocytes and hepatocyte.^105^

Liver sinusoid endothelial cells (LSECs) are fenestrated endothelial cells that form the lining of the hepatic sinusoids. The structure and function of LSECs changes upon aging, which in turn impacts on liver functions. Age-induced morphological changes in LSECs have been described in rodents and humans and are characterised by defenestration (defined by the decrease in the number and size of fenestrae), endothelial thickening, and basal lamina and collagen deposition in LSECs. It was recently shown that senescence markers increase in older mice, followed by an enhanced ability to clear macromolecular wast.^106^ However, this enhanced ability rapidly declines with further aging, probably due to increased endothelial thickness and senescence-induced silencing of scavenger receptors and endocytosis genes. Of importance, age-dependent changes in LSECs were recently confirmed in the livers of elderly humans, underscoring that aging and senescence are accompanied by significant liver sinusoidal dysregulatio.^107^

Kupffer cells (KC), the resident macrophages of the liver, are located within the lumen of the liver sinusoids. KCs are the key detector of commensal or pathogenic microbial signals, danger signals, and tumour cells moving through the hepatic circulation; they produce soluble cell mediators such as *TNFα* and IL-6 as part of the innate immune response. While there have been many studies on the effects of aging and senescence on macrophages, the effect on KCs has not been well characterised on a cellular and molecular level. The effects of aging on macrophages include reduced phagocytosis and autophagy and increased production of cytokines such as IL-6, suggesting that KCs might be one source of elevated IL-6 – a characteristic of old ag.^107^

Although the aforementioned studies imply that senescence is variously involved in NAFLD pathogenesis and progression, it is of interest whether data from animal studies can be translated to humans. In the next paragraph, we will elaborate on the evidence of a role of cellular senescence in NAFLD in humans.

## Clinical evidence linking ageing and senescence to NAFLD

Under normal circumstance, around 3–7% of hepatocytes are senescent. This percentage can increase to 50–100% in end-stage liver disease.^108^^,^^109^ As mentioned, senescence in the liver can have protective as well as deleterious effects on liver function and metabolism. Interestingly, hepatocytic senescence is considered to act as a protective mechanism against the development of HCC.^110^ Data obtained from studies in humans revealed that several hallmarks of cellular senescence are present in biopsies of individuals with NAFLD and that the number of senescent cells increases with disease progression. Relative nuclear size of hepatocytes in individuals with NAFLD was significantly larger than the normal value of healthy controls, independent of telomere length.^111^ Interestingly, telomere length correlated negatively with nuclear size in both individuals with NAFLD as well as in healthy controls, while the average nuclear size of the hepatocyte only correlated with age in the healthy controls. This suggests that nuclear enlargement proceeds independently of age in individuals with NAFLD. Other studies also showed that average telomere length in the livers of individuals with NAFLD is shorter than in age-matched healthy control.^111^^,^^112^ Moreover, in a longitudinal study of 6 years, it was shown that individuals who developed NAFLD had shorter telomeres in peripheral blood leukocytes at the end of the follow-up period compared to the individuals who did not develop NAFLD.^113^ Despite this observation, individuals who developed NAFLD were metabolically already more challenged compared to the individuals without NAFLD. Also, Laish *et al.*^114^ observed shorter telomeres in peripheral lymphocytes accompanied with a higher expression of telomerase reverse transcriptase messenger RNA compared to healthy controls. To what extent the telomere length in peripheral blood corresponds to telomere length in liver cells remain to be investigated. Nevertheless, these results support a role for telomere dysfunction in the development of NAFLD.

Bearing this evidence in mind, one might ask the question: how do telomeres signal senescence? It has been hypothesised that a protein complex that shapes and safeguards human telomeres, also called the “shelterin”, destabilises with each cell division.^115^^,^^116^ This destabilisation results in exposure of the telomere, which is subsequently recognised as a double-strand DNA break. This triggers recruitment of proteins belonging to the DDR such as ATM and H2AX and Rad17.^117^ As mentioned, the DDR activates transcription factors such as p53, which is a positive regulator of p21. Both *in vivo* and *in vitro* studies have shown that p21 plays a key role in telomere-induced senescence.^46^^,^^118^ Although the majority of studies provide evidence that senescence is a result of telomere shortening, several other reports now suggest that telomere dysfunction can also occur in a length-independent manne.^46^^,^^48^^,^^119^^,^^120^ For example, chronic mild inflammation is able to induce telomere gene damage in hepatocytes and enterocytes of the small intestine, irrespective of telomere length.^119^^,^^120^ Moreover, it has been suggested that DNA damage is more likely to occur at long telomeres as they represent a more abundant target for lesion formation which can explain length-independent DDR activation.^48^ Interestingly, the link between telomere-induced senescence and the p16 pathway is less clear and, compared to p21, the link between p16 expression and NAFLD in humans is not that robust.^32^

Several studies revealed a link between DNA damage in hepatocytes, hepatocytic senescence and NAFLD.^32^^,^^112^ Aravinthan and colleagues^112^ showed that DNA damage is increased in the livers of individuals with NAFLD and increases with disease progression (*i.e.* NASH and NAFLD with advanced fibrosis). Moreover, by using paired biopsies from 35 individuals, hepatocyte p21 expression was shown to increase with disease progression, whereas individuals with disease improvement exhibited decreased expression of hepatocyte p21. Thus, hepatocytic senescence is a marker of disease progression. In addition, another study reported that hepatocytic senescence is positively correlated with the progression of liver fibrosi.^109^

Recently, a prediction model based on epigenetic DNA methylation was introduced to measure human chronological and biological age. Using this model, it is possible to predict the normal aging rate based on methylation patterns.[121], [122], [123], [124] One of the algorithms for this model is the so-called Horvath Clock, which represents an epigenetic profile comprising methylation levels of 353 CpG dinucleotide sites.^122^ Of these 353 CpG sites, 193 positively correlate with age when hypermethylated whereas 160 negatively correlate with age when hypomethylated. To illustrate the time frame of the Horvath clock, the DNA methylation score of embryonic stem cells is approximately zero and increases rapidly during normal development. The validity of peripheral DNA methylation to accurately predict chronological age of different tissues including the liver has been confirmed in multiple studies.^122^^,^^125^^,^^126^ Moreover, the intrinsic rate of the epigenetic clock can be altered by metabolic diseases. Obesity for example is able to alter the epigenetic clock for the liver, but not of other tissue.^126^ Recently, Loomba and colleagues have shown, using the Horvath clock, that individuals with NASH demonstrate significant acceleration in their biological age.^125^ Enrichment analyses of the genes associated with differentially methylated CpG islands revealed significant enrichment of senescence pathways such as p53 signalling, suggesting that, in line with other reports, a specific pattern of DNA methylation is another senescence marker associated with NAFLD and its progression to NAS.^125^^,^^127^ Murphy *et al.* showed that individuals with NAFLD with mild fibrosis could be distinguished from individuals with NAFLD with advanced fibrosis based on different methylation patterns.^128^ Individuals with advanced fibrosis had more hypomethylated genes in their liver biopsies, resulting in overexpression of tissue repair genes, whereas metabolism-associated genes were hypermethylated, resulting in downregulation of these genes. Another study found that alterations in methylation patterns in genes involved in the cell cycle are closely related to oxidative DNA damage in the livers of individuals with NAFLD.^129^ Collectively, these data indicate that NAFLD may induce altered methylation profiles in a plethora of cells, including even peripheral blood cells. However, from this perspective, senescence is a consequence of the metabolic dysregulation and inflammatory phenomena occurring within the liver instead of a causal player.

## Treatment options for targeting senescence

Usually, NAFLD is accompanied by other obesity-induced age-related diseases. This inevitably leads to polypharmacy because most treatment strategies are disease specific. Unfortunately, polypharmacy can lead to adverse events, unpredictable drug interactions and poor adherence.^82^ Bearing in mind that senescent cells are present in several metabolic diseases, targeting senescent cells has emerged as an attractive therapeutic strategy to simultaneously treat these diseases.

Targeting senescence could be performed by inhibiting the SASP or by selectively eliminating senescent cells using senolytics. SASP inhibitors, also known as senomorphics, target signalling pathways that are involved in the regulation or exacerbation of the SASP, such as target of rapamycin complex 1 (mTORC1), JAK1/JAK2, STAT3, and mitochondrial dysfunctio.^82^ Senomorphics include rapamycin, ruxolitinib, glucocorticoids and metformin.[130], [131], [132] However, most of the senomorphics do not reduce the entire range of SASP factors and most have many other effects beyond their senomorphic properties. Therefore, disentangling the specific effects of SASP modulation on age-related phenotypes is challenging. In addition, senomorphics would need to be administrated continuously to maintain SASP suppression, which limits their applicability.

Senolytic agents were first discovered in 2015. Although it was known as early as 1995 that senescent cells are resistant to apoptosis,^133^ the authors hypothesised that senescent cells depend on senescence-associated antiapoptotic pathways (SCAPs), which permit senescent cells to survive their own SASP.^134^ Using a combination of bioinformatic tools and *in vitro* RNA interference studies, it was verified that senescent cells rely on SCAPs. Hence, SCAPs were identified as the Achilles heel of senescent cells. Since this discovery, considerable progress has been made in identifying small molecules, peptides and antibodies that selectively induce apoptosis in senescent cells. The combination of dasatinib, which is an FDA-approved tyrosine kinase inhibitor and the antioxidant quercitin, which is a flavonol present in many fruits and vegetables, successfully induced apoptosis in senescent cells *in vitro* and in rodent model.^133^ According to *in vitro* data, a brief disruption of pro-survival pathways is adequate to^82^^,^^135^ suggest that senolytics could be administrated intermittently, which would reduce the risks of adverse effects compared to continuous treatment.

As mentioned, administration of dasatinib plus quercitin successfully eliminated both adipocyte and hepatocytic senescence and decreased lipid accumulation.^33^ So far, no studies in individuals with NAFLD using dasatinib plus quercitin have been performed. However, several clinical trials addressing the efficacy of dasatinib plus quercetin to treat metabolic diseases are currently ongoing.^136^ Combining results from 2 human clinical trials using dasatinib plus quercitin for the treatment of diabetic chronic kidney disease and idiopathic pulmonary fibrosis – diseases characterised by the accumulation of senescent cells – revealed that eliminating senescent cells, which was confirmed by analysing adipose tissue, improved clinical outcomes.^137^^,^^138^ These clinical investigations have proven that the risks of using dasatinib plus quercitin in combination were minimal in relation to the clinical benefits. However, several senolytic drugs, including dasatinib, have been used for cancer treatment and often lead to adverse effects such as nausea, vomiting, diarrhoea and skin rashes when taken continuously. Moreover, senolytics also have other effects. For example, the antioxidant quercetin inhibits ferroptosis and can decrease inflammation and lipid metabolism, all pathways that are associated with NAFL.^80^^,^^81^ Although it has been suggested that senolytics could be administrated intermittently, thereby reducing the risks of adverse events, large clinical trials are needed to define the benefits and potential risks of these drugs.

## Conclusions

Data from studies in rodents and humans have shown that NAFLD is accompanied by an increase in senescent cells in the liver, and that the number of senescent cells is associated with a more advanced disease state. Despite the strong associations between senescence and NAFLD in humans and the work derived from *in vitro* studies and rodents, it remains to be determined if hepatic senescence is a mere consequence of the metabolic dysregulation and inflammatory phenomena in NAFLD or a causal player in the development of this disease. Although a causal role of cellular senescence must be further substantiated and subsequently established in humans, this pathophysiological process holds great potential, particularly when bearing in mind that there is currently no effective treatment for NAFLD. Targeting senescence has emerged as an attractive therapeutic target for NAFLD since senescence might be involved in the full spectrum of the disease (*i.e.* from early steatosis to cirrhosis). Moreover, senolytic drugs can be administrated intermittently, thereby minimising potential toxic effects and increasing adherence in the individual often affected by multiple morbidities and thus treated with multiple medications. Nevertheless, clinical trials conducted in individuals with NAFLD using senolytics have not been performed. Such trials are needed to better define the benefits and potential risks of these drugs. To increase efficacy and accuracy of these clinical trials, new or composite assays are needed, and development of these assays should be a top priority for the field.

## Financial support

M. Nieuwdorp is supported by a 10.13039/501100001826ZONMW-VIDI, Netherlands grant 2013 [016.146.327] and a grant from the Dutch Heart Foundation, Netherlands, CVON IN CONTROL. HH is supported by a Senior Fellowship (2019.82.004) of the 10.13039/501100003092Dutch Diabetes Research Foundation. JK received a VENI grant by 10.13039/501100001826ZONMW (91619098).

## Authors’ contributions

All authors substantially contributed to the conception, design and interpretation of the published literature cited in this review.

## Conflict of interest

The authors declare no conflicts of interest that pertain to this work.

Please refer to the accompanying ICMJE disclosure forms for further details.
